# Comparative Cancer Cell Signaling in Muscle-Invasive Urothelial Carcinoma of the Bladder in Dogs and Humans

**DOI:** 10.3390/biomedicines9101472

**Published:** 2021-10-14

**Authors:** Maria Malvina Tsamouri, Thomas M. Steele, Maria Mudryj, Michael S. Kent, Paramita M. Ghosh

**Affiliations:** 1 Veterans Affairs-Northern California Health System, Mather, CA 95655, USA; tmsteele@ucdavis.edu (T.M.S.); mmudryj@ucdavis.edu (M.M.); 2 Department of Urologic Surgery, School of Medicine, University of California Davis, Sacramento, CA 95718, USA; 3 Graduate Group in Integrative Pathobiology, University of California Davis, Davis, CA 95616, USA; 4 Department of Medical Microbiology and Immunology, School of Medicine, University of California Davis, Davis, CA 95616, USA; 5 Department of Surgical and Radiological Sciences, School of Veterinary Medicine, University of California Davis, Davis, CA 95616, USA; mskent@ucdavis.edu; 6 Department of Biochemistry and Molecular Medicine, School of Medicine, University of California Davis, Sacramento, CA 95718, USA

**Keywords:** urothelial carcinoma, bladder cancer, comparative oncology, basic & translational cancer research, naturally occurring models of cancer, canine cancer, molecular cancer therapeutics

## Abstract

Muscle-invasive urothelial carcinoma (MIUC) is the most common type of bladder malignancy in humans, but also in dogs that represent a naturally occurring model for this disease. Dogs are immunocompetent animals that share risk factors, pathophysiological features, clinical signs and response to chemotherapeutics with human cancer patients. This review summarizes the fundamental pathways for canine MIUC initiation, progression, and metastasis, emerging therapeutic targets and mechanisms of drug resistance, and proposes new opportunities for potential prognostic and diagnostic biomarkers and therapeutics. Identifying similarities and differences between cancer signaling in dogs and humans is of utmost importance for the efficient translation of in vitro research to successful clinical trials for both species.

## 1. Introduction

### 1.1. Bladder Cancer and Its Treatment in Human Patients

In 2021, it is estimated that approximately 65,000 men and 19,500 women will be diagnosed with bladder cancer (BlCa) in the US, with approximately 17,000 people succumbing to the disease [1]. Most BlCa cases arise in the urothelium (transitional epithelium) and can be classified histologically as urothelial carcinoma (UC) (also known as transitional cell carcinoma (TCC)) and can be either superficial (non-muscle invasive) or muscle-invasive (infiltrating muscle and the layers beyond). UCs are also classified as papillary (finger-like projections from the inner surface of the bladder toward the center) or flat. Other types of BlCa that are far less common include squamous cell carcinoma, adenocarcinoma and small cell carcinoma, as well as rare sarcomas—including soft tissue sarcoma and rhabdomyosarcoma.

While superficial disease is more prevalent and treatable, patients with this disease often experience recurrence with muscle invasive urothelial carcinoma (MIUC). The latter is likely to metastasize and often proves to be lethal; the 5-year survival rate of patients with metastatic disease is 5% [2]. Standard of care treatment for localized high grade BlCa in human patients involves cystectomy, i.e., the complete removal of the urinary bladder and lymph nodes, with or without the addition of neo-adjuvant chemotherapy or radiation therapy [3]. For patients with recurrent or metastatic UC, cisplatin-based combination chemotherapy regimens are the standard of care for first-line therapy in patients who can tolerate it [3]. These protocols include MVAC (methotrexate, vinblastine, doxorubicin, and cisplatin), CMV (cisplatin, methotrexate, and vinblastine) and GC (gemcitabine and cisplatin). For patients who are not candidates for cisplatin, carboplatin-based regimens have demonstrated comparable responses [4]. Immune checkpoint inhibitors against programmed death-1 (PD-1) or programmed death-ligand 1 (PD-L1) were shown to have activity in patients resistant to or ineligible for chemotherapy [5]. *Enfortumab vedotin*, an antibody drug conjugate that links the microtubule inhibitor monomethyl auristatin E (MMAE) to an antibody to Nectin-4, a cell adhesion protein expressed on bladder tumor cells, has been approved since 2019 for patients with metastatic BlCa that progressed after treatment with both platinum-based first-line chemotherapy and second-line therapy with an immune checkpoint inhibitor [6,7]. Finally, erdafitinib, a fibroblast growth factor receptor (FGFR) inhibitor, was approved for patients with FGFR mutations that have progressed after chemotherapy [8].

### 1.2. Canine Patients as Naturally Occurring Models of Human MIUC

Much of the current progress in human bladder cancer has been made possible by preclinical studies in vertebrate animals. The most commonly used in-vivo models of BlCa are mouse models, in which tumors need to be induced either with chemical exposure of the bladder or genetically engineering (immunocompetent animals) or xenograft formation with human cell lines either orthotopically, in the bladder wall, or heterotopically, in the flank (immunodeficient animals) [9]. However, in recent times, canine patients with urothelial carcinoma have emerged as a viable naturally occurring model of human BlCa [10,11]. Hence, a greater understanding of canine UC is warranted. This review compares the fundamental molecular pathways of canine UC to those of human UC and explains how treatment and research of canine UC has changed over time. Better understanding of the pathophysiological basis of canine UC will lead to the development of better diagnostic biomarkers and more effective therapeutic options for both dogs and humans. The overall goal will be to conduct additional preclinical phase 0 studies in canine UC patients that can be translated to phase 1 trials in humans.

## 2. Muscle-Invasive Urothelial Carcinoma in the Dog

MIUC is the most common type of urinary bladder malignancy in the dog, affecting greater than 50,000 dogs annually in the US [12] with a ratio of female: male dogs being approximately 1.8:1 [12]. This is in direct contrast to human patients where the ratio of female: male patients is approximately 0.3:1 [1]. Early diagnosis of this disease in the dog is often challenging due to presentation with non-specific clinical signs that resemble those of other lower urinary tract disease (LUTD), including bladder inflammation or infection as well as stones, crystals, or debris in the bladder or urethra. Common clinical signs include difficulty urinating, frequent attempts to urinate, blood in urine and concurrent bacterial infection [13]. MIUC is typically diagnosed late in the dog with >10% canine patients presenting with metastatic disease at the time of diagnosis [14]. Canine MIUC has poor clinical prognosis, partly due to the delay in conclusive diagnosis, and due to ineffective definitive therapeutic options [15].

### 2.1. Standard Treatment for MIUC in Dogs

Even though cystectomy is the standard of care for localized MIUC in humans [3] as stated above, this is not the case in canine MIUC for several reasons. Canine MIUC is usually located at the trigone area of the bladder (whereas human MIUC is located throughout the bladder), often projecting towards the urethra or the prostate gland, making it anatomically difficult to excise the tumor while maintaining a “negative” surgical margin (tumor-free perimeter at the area of the incision) [16]. Several procedures have been proposed for complete cystectomy and urinary diversion in dogs [17,18,19,20], but severe side effects along with excessive cost of the procedure render cystectomy an unattractive option for the canine patient. For tumors that extend through the urethra and block urine outflow, palliative options are available, such as tumor “debulking” through transurethral resection with electrocautery/ laser or placement of a urethral stent to restore urine potency but can cause urinary incontinence [21].

As a result, combination therapy with non-steroidal anti-inflammatory drugs (NSAIDS), with or without the addition of chemotherapeutics has been the standard of care for canine MIUC (Table 1). The most commonly used NSAID has been the cyclooxygenase (COX) -inhibitor piroxicam, but other drugs in this category have also been used as well, such as meloxicam, carprofen and deracoxib [16]. Chemotherapeutics that has been used in the clinic include carboplatin, cisplatin, mitoxantrone, doxorubicin, vinblastine and gemcitabine. Despite the different therapeutic protocols tried in the clinic, dogs often become resistant to therapy and the median life expectancy for canine MIUC is approximately 105 (90–120) days with single agent therapy and 205 (180–240) days when NSAID inhibition is combined with chemotherapy [16,21].

Even though radiation therapy is a standard therapy used in humans with MIUC, it has not been used as commonly in dogs with MIUC. Besides being less clinically available than in human medicine, external beam radiotherapy caused gastrointestinal adverse effects to the pelvic region, that were ameliorated by using more finely fractionated dosing schemes or by delivering a lower total dose [22,23] as well as using intensity-modulated radiation therapy [24]. The addition of radiotherapy to piroxicam and mitoxantrone was better tolerated by the animals, but it did not add a clinical advantage to the administration of these drugs without radiotherapy [25]. A pilot study in 4 dogs with MIUC involved the use of neoadjuvant chemotherapy (gemcitabine/piroxicam), external-beam radiation and adjuvant chemotherapy (carboplatin) with promising results [26].

Finally, in contrast to human MIUC, immune checkpoint inhibition is not part of standard-of-care treatment for canine MIUC. After establishing the efficacy of numerous anti-PD-1/PD-L1 antibodies for the treatment of human malignancies (including MIUC [27]), several monoclonal antibodies were developed against canine PD-L1/PD-1 [28,29,30]. PD-L1 protein expression was detected in 100% (20/20) of canine MIUC tissues [30], so PD-L1/PD-1 blockade holds promise in canine MIUC similarly to human MIUC. Clinical immunomodulation of canine MIUC was recently examined using mogamulizumab, an anti- CC chemokine receptor 4 (CCR4) monoclonal antibody [31]. Mogamulizumab was administered in combination with piroxicam (*n* = 14 dogs) and compared to 14 dogs treated with piroxicam alone. Administration of mogamulizumab and piroxicam increased overall survival [474 (≥259) and 241 (108–516) respectively] and progression-free survival [189 (91–397) and 76 (21–161) respectively] in comparison to treatment with piroxicam alone, holding promise for further exploring this area of immunotherapy in canine MIUC. Overall, there is the urgent need for more effective and tolerated therapeutic options in canine MIUC.

### 2.2. Similarities between Canine and Human MIUC

Several factors point to using MIUC in the dog as a naturally occurring model for human MIUC: predisposing risk factors, clinical presentation, pathophysiological characteristics, genetic and epigenetic regulation, metastatic behavior and response to chemo- and immunotherapies [10,11,32,33]. Dogs are immunocompetent animals that live in the same environment as humans, come in contact with millions of antigens daily and receive multiple vaccinations starting at a very young age [34]. Dogs are exposed to cigarette smoke, pesticides, and other chemicals that are known risk factors for human BlCa [9]. Therefore, they represent a more comparable naturally occurring model to humans than immunodeficient mice. not only for conventional chemotherapeutic approaches but also as models for immunotherapy [34]. Therefore, dog BlCa reflect human BlCa in studies of environmental risk factors and can partake in clinical trials involving the use of novel therapeutics prior to starting human clinical trials.

### 2.3. Differences between Canine and Human MIUC

There are also differences between dog and human BlCa that need to be kept in mind. In contrast to human BlCa where most cases are represented by low-grade, superficial UC, more than 90% of canine BlCa cases are intermediate to high grade MIUC [11]. Further, MIUC in humans can be found in various locations in the bladder, predominantly on the lateral bladder walls [35], whereas tumors in dogs are mostly located in the trigone area of the bladder, usually extending through the urethra (or the prostate for male dogs) [10]. There are several theories that could explain this phenomenon. First of all, this could be attributed to the different orientation of the bladder in dogs compared to humans [36] and therefore the “pooling” of urine in the trigone area in the former. In addition, studies in rodents to identify potential stem cell niches in the bladder, showed that slow-cycling, progenitor cells (EdU retaining) were concentrated in the trigone area, close to the urethra (although found throughout the bladder) [37]. Another study showed that cells harvested from the caudal area of the bladder (including bladder neck and trigone areas), had higher proliferative and clonogenic capacity than those harvested from the cephalic area, properties that could indicate stemness. In contrast, human patients are thought to shed renal cells into the bladder—these include stem cells (called urine-derived stem cells) that can insert themselves and self-renew at different parts of the bladder [38]. There also appear to be some demographic genetic differences—for example, more female dogs suffer from BlCa compared to male dogs—whereas in human patients it is the other way around. This may be related to the fact that most male dogs are castrated at a fairly young age and therefore would not respond to hormones as human patients would, perhaps providing a protective effect [39]. Therefore, dog patients will not follow hormone dependent aspects of human MIUC such as those recently reported on [39].

Despite these differences, there are several similarities in the signaling pathways that lead to MIUC development in both canine and human patients that make the use of canines in preclinical trials for MIUC more promising. Note that canine patients will not usually model non-muscle invasive BlCa (NMIUC) prevalent in human patients as very few dogs are diagnosed with non-muscle invasive disease; however, they can serve as good models for MIUC. This review will outline the most important pathways that regulate tumor initiation and progression in dogs with UC, identifying similarities and differences with the homologous pathways in human MIUC.

## 3. Cell Cycle Regulation and Evasion of Apoptosis

**Figure 1:**
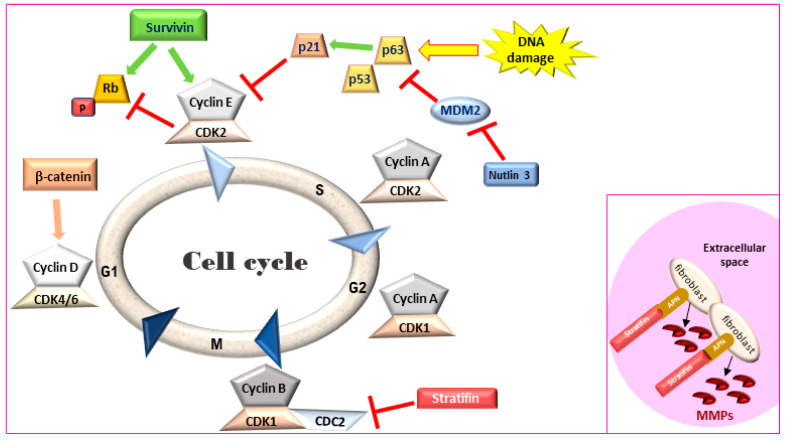
Cell cycle regulation. Survivin leads to an accelerated S phase and phosphorylates Rb thereby blocking its action. Stratifin blocks CDK1-Cyclin B complex causing G2/M arrest. **Inset:** In the extracellular space, stratifin can bind to aminopeptidase N (APN) on the plasma membrane of stromal fibroblasts and lead to the production of matrix metalloproteinases (MMPs). Rb: retinoblastoma, MDM2: mouse double minute 2 homology, CDK: cyclin-dependent kinase.

Maintaining a balance between cell proliferation and cell death is crucial for tissue homeostasis. Signaling for cell proliferation starts with the binding of extracellular growth signals to cell membrane receptors that are then transmitted to the nucleus through signal transduction pathways. In the nucleus, phosphorylation of a cascade of cyclins/CDK complexes (Figure 1) leads to cell cycle progression and cellular proliferation under the control of the tumor suppressor genes p53 and Rb [40]. However, if the conditions for cell division are not optimal, the tumor suppressors can induce cell cycle arrest and even condemn the cells to apoptosis if normal growth conditions are not restored or if cellular defects are irreversible [41]. The inactivation of these tumor suppressor genes represent the key molecular features of MIUC [42] and have been studied in detail in human disease [43]. Comparison of the process of cell cycle progression and arrest as well as apoptosis and survival demonstrate a closely related process in canine and human MIUC.

### 3.1. p53 Family of Proteins—The Master Regulator of Cell Cycle

p53, an extensively studied tumor-suppressor and transcription factor in cancer, is activated in response to cellular insult, stimulating transcription of genes related to cell-cycle regulation, cell-cycle arrest, repair and eventually apoptosis to prevent accumulation of damaged or malignant cells. However, in cancer, p53 is often lost or mutated and p53 mutation is correlated with advanced tumor stage and grade in human BlCa [44]. It is commonly understood that mutant p53 can function in a dominant negative manner to pervert the function of the wild-type p53 protein, which is transiently expressed in response to irradiation or other forms of DNA damage, and then rapidly degraded. Two homologues of p53, p63 and p73, belong to a family of related transcription factors [45]. In canine cancer, the role of p53, p63, and p73 was first studied in 2009 and revealed that the sequences of these transcription factors were 87%, 99.6% and 81% homologous to their human counterparts respectively [46]. Both the wild-type and the mutant form of p53 were discovered in different canine cell lines. In addition, the direct p53 target, p21 share a 80% similarity between human and canine amino acid sequence [46].

Nuclear p53 IHC expression was identified in 26% (5/19) of canine bladder tumors but not in normal bladder tissue [47]. The expression of p63 was found to be significantly lower in canine MIUC tumors than in tissues from dogs with polypoid cystitis or healthy dogs (*p* < 0.01). Lower expression of p63 in IHC was significantly associated with vascular infiltration (*p* < 0.05), presence of metastasis (*p* < 0.01) and shorter dog survival (*p* < 0.05) when compared to dogs with higher p63 expression. It was concluded that p63 could serve as biomarker for the prognosis of canine UC (Table 2) [48]. In 2018, pathway analysis by RNA-seq identified the p53 pathway to be significantly downregulated (bias-corrected z score = −2.977) in canine MIUC tumors versus normal bladder tissue [49]. Considering the importance of this pathway in tumor initiation and progression for both humans and dogs, the p53 pathway and associated mutations need to be further elucidated in canine MIUC.

### 3.2. Evasion of Apoptosis—The Role of Survivin

Survivin, a member of the inhibitors of apoptosis protein (IAP) family, is a regulator of cell division and proliferation and a suppressor of apoptosis. When survivin translocates to the nucleus, it leads to an accelerated S phase, CDK2/cyclin E activation and Rb phosphorylation (Figure 1) whereas survivin knockdown inhibits cell proliferation in a dose-dependent manner via cell-cycle arrest at the G2/M checkpoint and leads to apoptosis [50,51]. Although adult tissues express this protein in much lower levels [52], high survivin transcriptional and protein expression has been described in rapidly proliferating normal cells both during development and in tumors. Survivin expression in IHC was detected in human MIUC tumors (78%) but not in normal bladder tissue [53,54]. Survivin expression was correlated with higher histopathological grade, disease progression and poor overall survival [53,54,55,56], with strong nuclear staining correlated with a worse clinical outcome in human BlCa patients [57]. Survivin was detected in 100% of urine samples of human patients with MIUC but not in the urine of healthy individuals. Moreover, urinary survivin levels along with liquid-based cytology provided specificity and sensitivity of over 90% for human MIUC diagnosis [58,59].

Canine survivin mRNA and protein are more than 90% homologous to the human counterparts [60]. Survivin expression and subcellular localization in canine MIUC was first assessed in 2008 between MIUC tumors and normal bladder tissue [61] as well as between MIUC tumors, cystitis and normal bladder tissue samples [62]. Rankin et al. reported that the difference in mRNA and cytoplasmic protein levels of survivin between MIUC samples and healthy controls did not reach statistical significance (*p* = 0.06 and *p* = 0.07 respectively) [61], However, 68% of MIUC samples had nuclear survivin localization that was not detected in any of the normal bladder tissue samples (*p* < 0.001), supporting the immunohistochemical studies in human BlCa [63]. In an additional study [62], nuclear survivin was also detected in 50% of cystitis tissues whereas cytoplasmic survivin was only detected in 8% of these tissues. In 2017, a study comparing canine tumors of different origins (epithelial, mesenchymal and round-cell tumors) showed that survivin was significantly increased in malignant versus benign tumors (*p* < 0.05) at the transcriptional level [64]. Finally, in 2020, the survivin inhibitor EZN-3042 was shown to be well-tolerated in a phase I clinical trial of dogs with lymphoma, opening new avenues for the clinical targeting of this protein in veterinary oncology [65].

### 3.3. Stratifin

Stratifin (also named 14-3-3-σ and human mammary epithelial marker) has a dual role in cancer progression. When stratifin is localized inside the cell, it acts as a negative regulator of cell-cycle progression by causing a G2/M arrest and preventing the cdc2-cyclin B1 complex from entering the nucleus, which is required for cell-cycle progression through mitosis. When stratifin is released in the extracellular space, it can bind to aminopeptidase N (APN) on the plasma membrane of stromal fibroblasts and lead to the production of matrix metalloproteinases (MMPs), a group of proteolytic enzymes that alter the extracellular matrix, promoting cancer cell invasion and metastasis (Figure 1—inset) [66]. Stratifin protein expression is downregulated in human MIUC tumors as compared to normal bladder tissue [67]. Similarly, stratifin is overexpressed in the cytoplasm and nuclei of normal urothelial cells but lost in 53% of canine UC tumors. However, some cells in the invasive front of canine MIUC tumors showed increased cytoplasmic staining for stratifin and increased p53 levels [47].

### 3.4. Conclusions from the Comparative Analysis of Cell Cycle and Apoptosis Pathways

The above literature demonstrates that similar pathways that regulate cell cycle progression and cell survival are affected in both dog and human bladder cancer. Given that most chemotherapeutic agents work in cells that are rapidly proliferating or are cell cycle specific, these therapies show potential for both species.

## 4. Identifying the Urothelial Origin of Metastatic UC Cells-Uroplakin Family of Proteins

**Figure 2:**
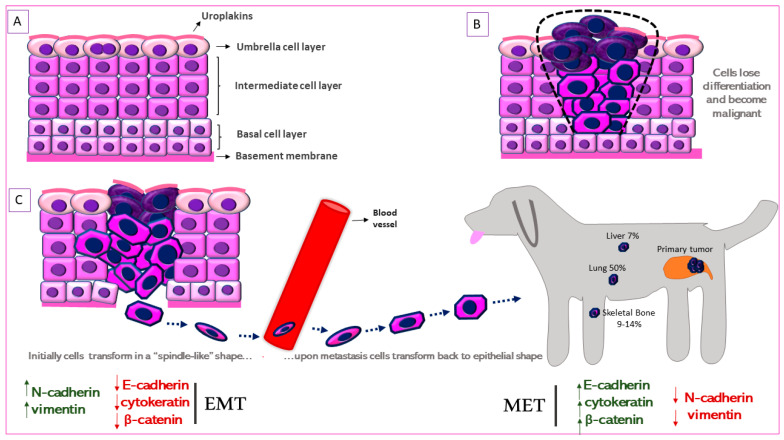
Schematic representation of Epithelial-to-Mesenchymal Transition (EMT) in canine MIUC. (**A**). Structure of normal urothelium. (**B**). Urothelial tumor outlined with the black dashed line. (**C**). EMT process in which the immotile urothelial cells upregulate mesenchymal (N-cadherin, vimentin) and downregulate epithelial (e-cadherin, cytokeratin) markers, acquire a “spindle-like” shape, become motile and infiltrate surrounding tissues and blood vessels. When they reach the metastatic site, the process is reversed (Mesenchymal-to-Epithelial Transition, MET) accompanied by the upregulation of mesenchymal and downregulation of epithelial markers. Some of the most common metastatic sites are depicted.

The urinary bladder wall comprises of five layers—the serosa, muscularis, submucosa, muscularis mucosa, and lamina propria (from outside to inside) [68]. The urothelium is a stratified layer of epithelial cells that covers the lamina propria (separated from it by a basement membrane) and consists of basal and intermediate cells with a superficial layer of “umbrella” cells that line the surface (Figure 2A) [69]. The umbrella cells are characterized by a highly specialized apical plasma membrane, the asymmetric unit membrane (AUM), which is a component of the permeability barrier that protects underlying tissues from noxious components of urine. The AUM is comprised mainly of four integral membrane proteins, the uroplakins (UP) Ia, Ib, II and III that form “plaques” on the surface of urothelial cells [70]. UPIa plays an important role in uropathogenic Escherichia coli (UPEC) pathogenesis while UPII and UPIII are type-1 transmembrane proteins that heterodimerize with UPIa and UPIb, respectively. UPIa and UPII appeared to be urothelium-specific, but UPIb was detected in several non-urothelial tissues [71]. UPII is a very specific marker for the identification of cells with urothelial origin of local or metastatic malignancies, and anti-uroplakin antibodies can potentially be used both for diagnostic and therapeutic purposes [72].

UPIII has a cytoplasmic domain that may function as a signal transducer. This integral membrane protein has been the gold-standard for identification of primary, anaplastic, cutaneous, subcutaneous and abdominal metastatic canine urothelial tumors [73,74,75]. Loss of tumor UPIII expression has been associated with higher tumor stage and grade and a metastatic phenotype in human BlCa (Figure 2B) [76]. On the other hand, urinary UPIII levels were significantly increased in human patients with BlCa compared to those with benign urological disease or healthy controls [77]. This indicates shedding of UPIII from the tumor into the urine. UPIII loss in BlCa is not confined to human patients but is seen in dogs with BlCa as well. Tumor classification was significantly associated with UPIII pattern (P = 1.49 × 10^−18^) as well as loss of UPIII (P = 1.27 × 10^−4^) in a study on a series of 99 canine proliferative urothelial lesions of the urinary bladder [78]. Furthermore, there were significant associations between depth of neoplastic cell infiltration into the bladder wall and overall UPIII pattern (P = 1.54 × 10^−14^), as well as loss of UPIII (P = 2.07 × 10^−4^) [78]. The expression of UPII and UPIII or loss thereof, may therefore be useful in both canine and human BlCa to identify cells of urothelial origin.

## 5. Cell Signaling Pathways of Canine MIUC

The pathways identified above refer to events that lead to significant changes in tumor growth (caused by increased proliferation), tumor regression (caused by apoptosis) and/or tumor progression (caused by invasion/metastasis). In most cases, these changes are triggered by alterations in signal transduction pathways resulting from mutations in key genes regulating these pathways. As discussed above, p53 loss or mutation is a common phenomenon in both human and canine BlCa. However, other pathways are also known to affect BlCa both in human and dog. We will consider here three classes of oncogenes that are known to affect these tumors.

### 5.1. Receptor Tyrosine Kinases

**Figure 3:**
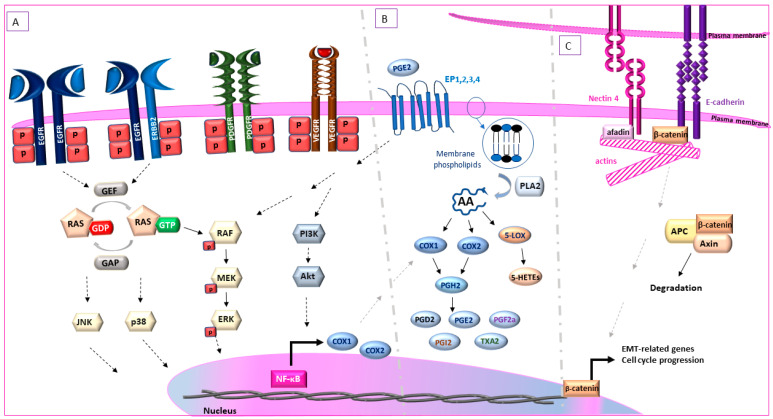
Fundamental signaling pathways governing canine UC. (**A**). Extracellular stimuli bind to membranous tyrosine kinase receptors such as PDGFR, VEGFR and the ErbB family, causing a phosphorylation cascade of the receptors’ tyrosine residues, leading to the activation of RAS-GTPase by GEF and subsequently the activation of RAF family of proteins. MEK is then phosphorylated by RAF which in turn phosphorylates and activates ERK. (**B**). PLA2 converts membrane phospholipids to AA. COX-1 and COX-2 convert AA to PGH2, which is a substrate for the synthesis of PGE_2_, PGD_2_ PGF_2a_, PGI_2_ and TXA_2_. 5-, 8-, 12- and 15- LOX convert A to 5-, 8-, 12- and 15-HETEs respectively. (**C**). Nectins are connected to actin cytoskeleton through the protein afadin. The extracellular domains of e-cadherins interact with the corresponding extracellular domains of E-cadherin molecules of adjacent cells, forming a “zipper”-like structure. The cytoplasmic domain of e-cadherin is indirectly associated with the cytoskeleton through binding to β-catenin. In the absence of Wnt signaling, β-catenin is ubiquitinated and degraded after forming a protein complex with adenomatous polyposis coli (APC) and axin. EGFR = endothelial growth factor receptor, ErbB2 = erythroblastic oncogene B 2, PDGFR = platelet-derived growth factor receptor, VEGFR = vascular endothelial growth factor receptor, NF-κB = nuclear factor kappa B, EP2 = prostaglandin E2 receptor 2, PG: prostaglandin, COX = cyclooxygenase, TXA2 = thromboxane A2, PI3K = phosphatidylinositol-3-kinase, Akt = protein kinase B, AA = arachidonic acid, GEF = guanine nucleotide exchange factor, GAP = GTP-ase activating protein, GTP = guanosine-5-triphosphate, GDP = guanosine-5-diphosphate.

The tyrosine kinase receptor family consists of the fibroblast growth factor receptor (FGFR), platelet-derived growth factor receptor (PDGFR), vascular endothelial growth factor receptor (VEGFR), the stem cell factor receptor (KIT), and most prevalently the EGFR (epidermal growth factor receptor) family of receptors (EGFR/HΕR1, ErbB2/HER2, ErbB3/HER3, ErbB4/HER4) [79]. These receptors consist of an extracellular, a transmembrane and an intracellular domain and, upon activation, they form homo- or heterodimers that interact with intracellular mediators, activating signaling pathways such as MAPK/ERK, PI3K/Akt and COX2 regulating cell proliferation, survival and metastasis (Figure 3A) [80].

#### 5.1.1. Fibroblast Growth Factor Receptor

The family of FGFRs consist of four members (FGFR1, FGFR2, FGFR3, FGFR4). While normal urothelium expresses very low levels of FGFR3, this receptor is overexpressed in certain bladder tumors [81] and has been considered a characteristic of low-grade non-muscle invasive UC with papillary morphology in humans [82]. FGFR3 can be also overexpressed in a small percentage of high-grade human MIUC cases [81]. In fact, FGFR mutations are present in approximately 20% of human patients that have relapsed after treatment with neoadjuvant chemotherapy and FGFR inhibition was approved in 2019 for metastatic MIUC cases that overexpress FGFR and have relapsed after chemotherapy [8].

Urinary FGF levels were significantly higher in dogs with MIUC (*n* = 7) compared to those with UTI (*n* = 10) or healthy dogs (*n* = 17) [83]. However, treatment with the nonsteroidal anti-inflammatory drug piroxicam, which is considered a standard therapy for dogs with BlCa, led to a decrease in urinary basic FGF (bFGF) levels (77% of treated dogs), and this decrease positively correlated with the reduction in tumor size [84]. The same group later showed that piroxicam, in combination with cisplatin, showed partial remission in 66% of the dogs, but the mechanism by which piroxicam affects urine bFGF levels is not known [84]. These studies indicate a major role for bFGF in the mediation of the tumor suppressive effects of piroxicam in canine patients and suggest future benefits of using FGFR inhibitors in dogs with BlCa.

#### 5.1.2. ErbB Family of Receptors

EGFR and HER2 are well-known oncogenes and targets for therapy in humans and are overexpressed in many different cancer types. HER2 and EGFR amino acid sequence in canine mammary carcinoma had 92% and 91% homology with the human counterpart, respectively, and carried the binding sites of trastuzumab (anti-HER2) and cetuximab (anti-EGFR) monoclonal antibodies [85], suggesting the importance of developing homologous “caninized” antibodies to be used in canine patients.

In humans, EGFR expression is significantly higher in MIUC [31/56 (55.4%)] compared to normal bladder tissue [1/10 (10%), *p* < 0.05] [86]. Similarly, EGFR transcriptional levels were assessed, through real-time PCR, between healthy dogs (*n* = 3) and dogs with MIUC (*n* = 4) while protein translation was assessed through immunohistochemistry (IHC), between dogs with MIUC (*n* = 25), dogs with polypoid cystitis (*n* = 5) and healthy dogs (*n* = 5). EGFR overexpression in UC was statistically significant compared to normal bladder with respect to mRNA (*p* < 0.05) and protein (*p* < 0.001) [87]. Gene expression profiling analysis has shown that the epidermal growth factor-EGFR pathway is enriched in canine MIUC tumors (*n* = 18) compared to age-matched normal bladder tissue (*n* = 4) (*p* < 4.65E-05, FDR > 2). In-situ EGFR IHC analysis confirmed these results, as EGFR was detected in similar percentages for human MIUC (73% and 79% respectively) [88,89].

Although HER2 does not have a ligand binding domain and must heterodimerize for activation, it is also the tyrosine kinase receptor with the strongest catalytic activity [80,90]. HER2 overexpression is associated with a more aggressive phenotype in human breast cancer, and treatment with the anti-HER2 monoclonal antibody trastuzumab has achieved response rates of up to 60% in the clinical setting [91]. HER2 overexpression was recently identified in 60% of canine MIUC [15] which is an even higher percentage than that seen in canine mammary tumors (22%) [92]. Further, transcriptome sequencing identified the HER2-encoding gene, ERBB2, as the second most highly upregulated gene in canine MIUC [49]. However, no correlation was found between HER2 levels and tumor stage in canine MIUC [15].

#### 5.1.3. ErbB Receptors in UC Diagnosis

Late diagnosis of canine MIUC is partly attributed to similar clinical signs between malignant and non-malignant low-urinary tract diseases, as mentioned above. EGFR IHC staining was able to differentiate between dogs with MIUC and polypoid cystitis (*p* < 0.001) with a sensitivity of 72% and specificity of 100%. Therefore, EGFR IHC expression could potentially assist in canine MIUC diagnosis [87]. In another study, strong HER2 expression was observed in 14/23 (60%) of canine MIUC tumors but not in normal bladder tissues [15]. Finally, although EGFR and ErbB2 have been studied extensively, ErbB3 has not been well studied in canine or human MIUC. ErbB3 is mainly researched indirectly through or along with EGFR and ErbB2. However, our lab is currently testing the effects of ErbB3 inhibition on cisplatin-resistance.

#### 5.1.4. Other Tyrosine Kinase Receptors

PDGFR and VEGFR are other important receptors involved in tumor neo-angiogenesis as reviewed elsewhere [93]. VEGFR1 and VEGFR2 mRNA levels were significantly increased in human bladder tumors as compared to normal bladder tissues (*p* < 0.02 and *p* < 0.001 respectively). In IHC studies VEGF and VEGFR1 expression was higher in human non-MIUC than MIUC whereas the opposite pattern was observed for VEGFR2 (*p* < 0.001) [94]. PDGFR-α was overexpressed in 62% of human bladder tumors and co-expression of PDGFR-α with c-met and Axl was correlated with poor patient survival (*p* < 0.01) [95].

In 2003, SU11654, a multi-tyrosine kinase receptor inhibitor (TKRI) (including PDGFR, VEGFR, KIT and Flt3) was administered in a phase I clinical trial of 57 dogs with various malignancies, including MIUC (*n* = 4) [96]. SU11654 caused partial to complete response in multiple dogs with multiple malignancies and resulted in stable disease in 3/4 dogs with MIUC. In 2017, expression level of PDGFR-β, VEGFR-2 and KIT were compared between canine MIUC tumors, cystitis samples and normal bladder tissue using IHC [97]. PDGFR was expressed in canine MIUC (100%), cystitis (90%) and normal (100%) bladder tissues, but PDGFR-β immunohistochemical expression was significantly elevated in MIUC compared to non-neoplastic bladder tissues (*p* < 0.0001). All samples stained positive for VEGFR-2. MIUC tumors had mostly moderate (40%) to intense (40%) staining and cystitis tissue samples showed mostly intense staining (60%). However, normal bladder tissue samples had mostly moderate staining (60%). KIT staining was observed in 36% of canine MIUC samples but was not detected in cystitis or normal bladder tissue.

In 2018, another study examined the effect of two RTKIs, axitinib (PDGFR, VEGFR, c-Kit inhibitor) and AB1010 (PDGFR and c-Kit inhibitor), in human and canine UC cell lines. Both RTKIs induced dose-dependent reduction of cell proliferation and apoptosis. In fact, there was a positive correlation between the reduction of cell proliferation and PDGFR and KIT protein levels. TKRIs increased the levels of COX2 and PGE_2_, effects that were blocked by the simultaneous administration of the selective anti-COX2 non-steroidal anti-inflammatory drug, indomethacin. The combination of these drugs did not affect nuclear factor kappa-light-chain-enhancer of activated B cells (NF-κB) levels, but decreased Akt levels and showed a greater effect in decreasing cell viability [79].

### 5.2. Arachidonic Acid Metabolism-Cyclooxygenases and Lipooxygenases

Cyclooxygenases (COX), COX-1 and COX-2 (or prostaglandin endoperoxidase synthases 1 and 2) represent the rate-limiting enzymes in prostanoid synthesis by converting arachidonic acid into prostaglandin H2. Prostaglandin H2 is a substrate for the synthesis of prostaglandins and thromboxanes, with PGE_2_ being the prevalent prostaglandin produced from COX-2 in cancer cells (Figure 3B) [98,99,100]. COX-2 is overexpressed in human MIUC, and COX-2 inhibition induced cell death via apoptosis and reversal of epithelial-to-mesenchymal-transition in human UC cell lines, thereby reducing the cell migratory potential [101]. Lipooxygenases (LOX) represent the alternative pathway for arachidonic acid oxidation by converting arachidonic acid to hydroperoxyeicosatetraenoic acids (HETEs). 5-, 8-, 12- and 15- LOX lead to the production of 5-, 8-, 12- and 15-HETEs respectively (Figure 3B). Strong expression of 5- and 12-LOX in IHC has been shown in human MIUC tissues and LOX inhibition resulted in apoptosis and dose-dependent growth inhibition in human UC cell lines [102].

#### 5.2.1. COX Inhibition—The “Gold” Standard Therapeutic Strategy in Canine UC

Treatment of canine MIUC with COX inhibitors as a monotherapy or in combination with other chemotherapeutics has been established for decades, due to favorable patient outcomes and limited toxicity, but the exact mechanism behind the anti-tumor effects of these drugs had not been well-elucidated [12,14,103]. COX-1 is expressed both in neoplastic and non-neoplastic tissue whereas COX-2 is overexpressed only in inflammatory bladder tissue of dogs [78] and in neoplastic bladder tissue of both dogs and humans [104,105]. Consequently, COX-1 inhibition is associated with gastro-intestinal, renal and blood coagulation side-effects. Despite the potential side effects of COX-1 inhibition, piroxicam, a non-selective COX inhibitor, has been the “gold-standard” in canine UC management [12,14,84,106] alone or in combination with other chemotherapeutics [84,107,108].

To minimize the side-effects associated with COX-1 inhibition, a selective COX-2 inhibitor is an attractive alternative, and in 2011, the anti-tumor effects of a selective COX-2 inhibitor, deracoxib, was used as a monotherapy for dogs with UC [109]. In 2012, the same group assessed the anti-tumor effects of another selective COX-2 inhibitor, firocoxib, alone or in combination with cisplatin and response was compared with cisplatin as a monotherapy for dogs with UC. Dogs receiving firocoxib alone showed the lowest overall toxicity followed by the combination of the drugs, with the highest toxicity observed in dogs treated with cisplatin alone. Firocoxib enhanced the anti-tumor potency of cisplatin since simultaneous administration of the drugs provided a longer progression-free survival compared to dogs that received cisplatin alone. This effect might have been greater, but cisplatin treatment had to be discontinued in the majority of dogs due to high toxicity. Finally, the administration of firocoxib alone showed favorable results, similar to those observed with piroxicam treatment but without the usual adverse effects of non-selective COX inhibitors [110].

In 2019, the anti-tumor properties of mavacoxib were assessed in a panel of canine cancer cell lines. Mavacoxib is a selective COX-2 inhibitor used for the chronic treatment of canine osteoarthritis. Exhibiting a long half-life (~15 days), it needs to only be administered once every 30 days, making it a unique NSAID that is suitable for chronic administration. Treatment with mavacoxib reduced proliferation, cancer cell viability, and cancer stem cell viability while inducing caspase-independent apoptosis in canine cancer cell lines in a dose-dependent manner independently of COX-2 expression levels [111]. Based on in vitro studies, mavacoxib represents a promising drug for clinical trials in dogs with MIUC and other COX-2 overexpressing malignancies. 

As mentioned above, this study had an interesting finding; the anti-tumor effects of mavacoxib were independent of COX-2 expression levels [111]. This outcome was supported by a previous study in which COX-2 and PGE_2_ levels were not directly correlated with the subsequent response to piroxicam treatment [112]. Even though, it has been proposed that distinguishing tumors with high-COX-2 expression in vivo and in vitro [113] could potentially determine which animals would respond to anti-COX treatments, COX-2 expression levels do not always correlate with the response to COX inhibition. In fact, studies have shown COX-2 independent effects of COX inhibitors in human malignancies [114]. Considering the importance of this pathway in canine MIUC, similar studies should be done in dogs to address potential COX-2 independent pathways that are affected by anti-COX treatment.

#### 5.2.2. Cyclooxygenase Signaling and Multi-Drug Resistance

- Prostaglandin E_2_ (PGE_2_)

An association has been reported between PGE_2_ expression levels and chemoresistance, as PGE_2_ signaling inhibition attenuated cancer stem cell “repopulation” after chemotherapy in a human BlCa xenograft [115]. High PGE_2_ expression levels have been reported in multiple canine cancers, including canine MIUC [116]. PGE_2_ exerts its biological action through four receptors, prostaglandin E_2_ receptor 1 (EP1), EP2, EP3, and EP4. The previously mentioned transcriptomic sequencing analysis identified PTGER2, the gene encoding EP2, to be the most differentially expressed gene in the canine MIUC tumors examined [49], indicating the crucial role of this pathway in canine MIUC pathophysiology.

- P-glycoprotein (P-gp)

Another mechanism of COX-2-inducible resistance to chemotherapy and prevention of apoptosis is through the upregulation of p-gp [106]. P-gp is an efflux “pump” belonging to the family of ATP-binding cassette (ABC) transporters and encoded by the multi-drug resistance (MDR1 or ABCB1) gene. It is located on the cell membrane and reduces the intracellular concentration of chemotherapeutics by pumping them out of the cell [117]. Interestingly, experiments in human BlCa cell lines showed that, fully functional p-gp can be transferred from drug-resistant to drug-sensitive cells. The degree of transfer proportionally increased with the duration of co-culture even though the cells were not in direct contact [117,118]. By IHC, P-gp levels were significantly higher in patients with MIUC than in healthy controls (*p* < 0.01) and associated with MDR. Finally, high p-gp transcriptional levels were negatively correlated with the patients’ overall survival [119,120].

The COX-2/PGE_2_ axis directly upregulates p-gp and breast cancer resistance protein (BCRP) transporter levels, and COX-2 inhibition was shown to increase the intracellular concentration of the chemotherapeutic agent mitomycin C in human BlCa cell lines [106,121]. Accordingly, p-gp was expressed in 40/52 (76.9%) of canine UC tumors, and in IHC, p-gp expression levels were significantly correlated with COX-2 expression (*p* = 0.043). This was not the case for multi-resistant protein (MRP), another member of the ABC transporter family of proteins, that was expressed in less than 20% of the tumors examined [122]. No statistical correlation was performed between p-gp expression levels and patient outcome, however there are multiple reports in other canine cancers. For instance, in high-grade canine lymphomas, p-gp expression was associated with poor clinical outcome as it was inversely correlated with overall survival (*p* = 0.012) and remission (*p* < 0.001) [123].

It should be noted that several dog breeds are genetically deficient in p-gp [124,125]. Although ABC transporter activity plays a crucial role in mediating chemo-resistance in canine malignancies, it should be approached with caution. There is evidence in both human and veterinary medicine that p-gp inhibitors can significantly increase the intracellular concentration of chemotherapeutics and other drugs, leading to severe toxicity [126].

#### 5.2.3. LOX Inhibition in Canine MIUC

5-LOX expression levels were similar between canine prostatic carcinoma and benign hyperplasia [127], but it is present in 65% of canine osteosarcoma with LOX inhibition reducing cell proliferation in-vitro [128]. Treatment with a dual COX/5-LOX inhibitor, tepoxalin, led to oxidative damage, induced apoptosis and exhibited a synergistic effect with doxorubicin in reducing cell proliferation in canine osteosarcoma cell lines [129]. 

Very little work has been done in characterizing LOX inhibition as a therapeutic strategy in canine MIUC. In 2019, 5-LOX levels were assessed with IHC in canine MIUC tissue for the first time, comparing cystitis and normal bladder tissue and checking for correlation with the presence of COX enzymes. COX-1 was expressed in 100% of normal bladder tissue samples whereas 5-LOX and COX-2 was only expressed in 10%. Cystitis samples were either positive for 5-LOX (23%) or COX-2 (31%) but not for both. Finally, 5-LOX and COX-2 was expressed in 95% and 90% of canine MIUC tumors [130]. However, more studies are needed to assess the importance of targeting COX-2/5-LOX in canine MIUC.

### 5.3. Nectin 4 in Cell Adhesion, Migration and Invasion

Nectin 4 belongs to the nectin family of cell-cell adhesion proteins [131]. Nectins are connected to the actin cytoskeleton through the protein afadin and strengthen cell-to-cell junctions with or without the recruitment of cadherins [132] (Figure 3C). Nectins participate in the process of metastasis as they are involved in cell movement, differentiation [132], invasion, and angiogenesis [133]. Nectin-4 is detected in low levels in certain normal human tissues (bladder, breast, stomach, esophagus, skin and salivary glands); however, it is upregulated mostly in the cell membrane and cytoplasm of human bladder, breast, ovarian, lung and pancreatic cancer tissues, with bladder and breast cancer tissues having the highest expression levels [7]. Membranous nectin-4 can be cleaved by ADAM10/ADAM 17 proteases [134] and serum circulating levels of nectin-4 have been associated with the presence of metastases [135,136] and worse patient prognosis [135,137,138,139]. Nectin-4 has been also described as a marker of cancer stem cells (CSC) and EMT, induced by Wnt/β-catenin signaling [140] as well as anchorage-independent cell proliferation in human breast cancer [141]. 

As mentioned earlier, an antibody drug conjugate targeting Nectin-4, has been approved since 2019 for human patients with metastatic MIUC that have progressed after treatment with both first- and second-line therapeutics [6]. As far as canine MIUC is concerned, the expression of nectin-4 has been shown in canine urothelium among other tissues [142]. In 2020, the expression of nectin-4 was also detected in the canine TCC-NU1 MIUC cell line. Nectin-4 targeted immunotherapy (oncolytic virotherapy using a recombinant measles virus) caused a dose-dependent reduction of cell viability in vitro (*p* < 0.01), as well as reduction in tumor growth in a mouse xenograft model, vivo (*p* < 0.05) [143].

### 5.4. Mitogen-Activated Protein Kinase (MAPK)/Extracellular Signal Regulated Kinase (ERK) Signaling Pathway

One of the hallmarks of cancer is signal-independent sustainable proliferation via independent activation of signaling pathway intermediates irrespective of extracellular stimuli [41]. Normally, extracellular stimuli bind to membranous tyrosine kinase receptors such as PDGFR, the ErbB family, and FGFR [144] initiating signaling to MAPK/ERK. Ligand binding causes a phosphorylation cascade of the receptors’ tyrosine residues, RAS and subsequently the RAF family of proteins. MEK is then phosphorylated by RAF which in turn phosphorylates and activates ERK, leading to the activation of genes related predominantly to cell cycle regulation and survival (Figure 3A) [144]. However, in cancer, the activation of the MAPK/ERK pathway can be self-regulated, independently of extrinsic stimuli [145], due to mutations in different intermediates of the signaling cascade.

#### 5.4.1. BRAF^V595E^ Mutation as a Diagnostic Biomarker in Canine UC

In 2015, a BRAF^V595E^ point mutation was discovered in canine MIUC [146] with a valine substitution of glutamic acid at codon 595 of the canine BRAF gene [147], homologous to the tumorigenic human BRAF^V600E^ mutation [148]. BRAF belongs to the RAF family of proteins that act as intermediates of the mitogen-activated protein kinase (MAPK)-extracellular signal regulated kinase (ERK) pathway. Therefore, aberrant RAF signaling can cause dysregulation of cell growth, survival, proliferation, differentiation, division, adhesion and apoptosis via MAPK/ERK activation [145,149]. Recently, a commercially available diagnostic test was developed to non-invasively detect the BRAF^V595E^ mutation in cells shed into the urine using droplet PCR. However, this mutation is only present in the urine of ~65–80% of dogs with MIUC [150,151], prostate cancer [151] and other types of canine cancers [145,152] making it non- specific for UC. In addition, the presence of the BRAF^V595E^ mutation might be breed specific. A study examining MIUC tissues from dogs of different breeds found that the presence of the BRAF^V595E^ mutation is more frequent in terriers (*n* = 15, 73%) compared to non-terriers with MIUC (*n* = 50, 36%) (*p* < 0.05) [152]. 

In 2020, an RNA sequencing experiment was performed on canine MIUC tumors to identify differentially expressed genes based on the presence (*n* = 11 tumors) or absence of the BRAF^V595E^ mutation (*n* = 4 tumors) as compared to normal bladder tissue (*n* = 5). Indeed, the presence of the mutation separated the tumors into two distinct clusters of differentially expressed genes [153]. Finally, the BRAF^V595E^ mutation can also be detected in circulating tumor cells in plasma samples of dogs with MIUC, showing its potential as a biomarker in another biofluid as well [154].

#### 5.4.2. BRAF^V595E^ Mutation as a Therapeutic Target in Canine MIUC

A comparative study between human (UM-UC-3, T-24, 5637) and canine (K9TCC#1Lillie, K9TCC#5Lilly) UC cell lines showed that the latter exhibited higher ERK phosphorylation than their human counterparts [79]. In 2019, the BRAF^V595E^ mutation was studied with respect to tumor progression and as a potential therapeutic target in different canine UC cell lines [155]. Measurement of the mRNA expression levels of target genes downstream of the MAPK/ERK pathway through microarrays indicated the constitutive signaling of this pathway in canine MIUC. Treatment with BRAF inhibitors (vemurafenib or PLX7904) or MEK inhibitors (selumetinib or trametinib) alone, showed a reduction in ERK 1 and 2 phosphorylation after 6 h in BRAF mutant canine UC cell lines that was increased again after 24 h. Conversely, treatment with BRAF inhibitors lead to the activation of the MAPK pathway in KRAS mutant angus1 and BRAF/KRAS wild-type cells. Similarly, inhibitors of wild-type RAF activate RAF signaling by increasing the formation of homo- and hetero-dimers and membrane translocation under the influence of RAS-GTPase activation. This contradictory activation leading to potential MEK/ERK phosphorylation and tumor growth is known as “paradoxical activation” [156]. The simultaneous pan-ErbB and MAPK/ERK inhibition with sapatinib and PLX7904, respectively, synergistically decreased cell viability in BRAF^V595E^ mutant canine UC cell lines [155]. Finally, in 2020 an association was found between MAPK/ERK and COX2/PGE2 pathways. Treatment with BRAF (dabrafenib), pan-RAF (LY3009120), MEK (PD0325901) and ERK (SCH772984) inhibitors caused a dose dependent decrease of COX-2/PGE2 levels. P38 and JNK pathways were also involved in the process as their pharmacologic inhibition led to a decrease in PGE_2_ levels [157]. 

## 6. Metastasis in Canine Urothelial Carcinoma: Epithelial-to-Mesenchymal Transition

Next, we compare the process of metastases in dog and human MIUC patients. As stated above, at the time of canine MIUC diagnosis, about 15% of dogs already experience distant metastases. The most common sites of metastases include lymph nodes (42%), lung (12.4%), liver (7%), abdominal wall (10%), skeletal bone (9–14%) and others [75,158,159] (Figure 2). Better elucidation of the pathophysiological mechanisms of metastasis in canine MIUC will lead to more efficient prevention of tumor progression. Here, we compare the mechanisms that are prevalent in canine and human metastatic BlCa.

In BlCa, epithelial-to-mesenchymal transition (EMT) is a phenomenon during which the immotile urothelial cells, that are strongly attached to the basement membrane, gain a “mesenchymal-like” phenotype. These cells acquire migratory properties, become motile and can infiltrate surrounding tissues while evading immune response. The mesenchymal-like cells metastasize to distant organs, where the reverse process, mesenchymal-to-epithelial transition (MET) occurs and enables the cells to adhere and form metastatic tumors. At the molecular level, EMT is characterized by the downregulation of epithelial markers, such as E-cadherin and cytokeratin, and upregulation of mesenchymal markers, like N-cadherin and vimentin (Figure 2C). EMT has been excessively studied in both human and canine BlCa and has been associated with disease progression and metastasis [160]. 

E-cadherins are single-pass transmembrane proteins that constitute the major component of anchoring junctions. The extracellular domains of E-cadherins interact with the corresponding extracellular domains of E-cadherin molecules of adjacent cells, forming a “zipper”-like structure (Figure 3C) [161]. The cytoplasmic domain of E-cadherin is indirectly associated with the cytoskeleton through binding to β-catenin and other proteins responsible for maintaining cellular adherence. In the absence of Wnt signaling, β-catenin is ubiquitinated and degraded after forming a protein complex with adenomatous polyposis coli (APC) and axin (Figure 3C). However, upon Wnt signaling activation or E-cadherin downregulation, β-catenin translocates to the nucleus and activates genes that lead to cell proliferation, progression and metastasis [162]. Downregulation of E-cadherin is also associated with downregulation of β-catenin and upregulation of vimentin. Furthermore, vimentin expression is associated with higher tumor stage and grade in human bladder tumors [163]. 

Accordingly, strong membranous β-catenin staining in IHC was observed in normal canine urinary bladder (*n* = 5) and cystitis (*n* = 5) specimens, whereas staining intensity in localized canine UC tumors (*n* = 25) was significantly lower (*p* < 0.05) [48]. Moderate vimentin protein expression has been reported in canine UC cell lines [164], and vimentin was expressed in the invasive front of canine UC tumors (*n* = 19). As expected, vimentin was also strongly expressed in cells of mesenchymal origin (bladder muscle layer, vascular endothelial cells, fibrocytes and adipocytes) in UC tumors and normal bladder tissue samples. In addition, vimentin co-localized with stratifin in cells at the invasive front of the MIUC tumors [47]. Different cellular localization of cytokeratin 7, UPIII and COX-2 (more details provided later) expression in IHC was observed between normal, inflammatory and neoplastic canine bladder tissue. A reduction of cytokeratin 7 and UPIII was observed in the more infiltrative high-grade carcinomas, possibly due to EMT [78]. This information demonstrates a central role for EMT in promoting metastasis in both human and dog BlCa patients. 

## 7. Metabolic Regulation of Canine MIUC

**Figure 4:**
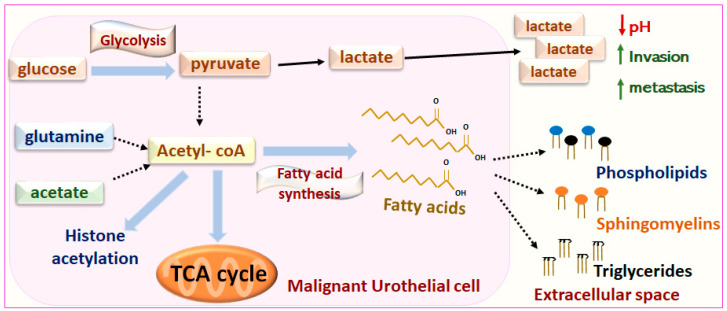
Metabolic reprogramming of tumor cells. Tumor cells convert glucose to lactate through aerobic glycolysis, even if sufficient oxygen levels are present. Lactate is then secreted to the extracellular space, acidifying the tumor microenvironment and favoring tumor invasion and metastasis. Tumor cells exhibit increased fatty acid production through acetyl-coA either from glucose, glutamine or acetate catabolism. Fatty acids are used to synthesize triglycerides, phospholipids and sphingomyelins. Acetyl-coA fuels the TCA cycle and contributes to epigenetic modifications through histone acetylation. Acetyl-coA: Acetyl- coenzyme A, TCA: Tricarboxylic acid.

Altered metabolic regulation has been considered a hallmark of cancer for the past decade [165]. In 1929 Otto Warburg first noticed that tumor cells converted glucose to lactate through aerobic glycolysis, even if sufficient oxygen levels were present. Lactate is then secreted to the extracellular space, acidifying the tumor microenvironment and favoring tumor invasion and metastasis (Figure 4) [166]. Metabolic competition between tumor cells and tumor infiltrating T lymphocytes can lead to weakened anti- tumor immune responses [167]. In addition, newly developing cancer cells require a vast amount of lipids for membrane construction and energy production as fatty acid oxidation produces more than twice the amount of energy as glucose catabolism. Therefore, tumor cells exhibit increased fatty acid production through acetyl-coA either from glucose, glutamine or acetate catabolism. Fatty acids conjugate with glycerol with or without phosphate to synthesize triglycerides and phospholipids, while conjugation with phosphocholine and sphingosine occurs to synthesize sphingomyelins [168]. Sphingomyelin synthesis, storage and catabolism are regulated by a negative feedback loop [169]. Furthermore, metabolic reprogramming also contributes to the epigenetic regulation of genes through the addition of acetyl or methyl groups [170] that act as “switch on/off” regulators of gene expression.

### Metabolomic-Lipidomic Analyses for Biomarker Discovery

Metabolomic analyses profile the end-products of gene expression and cell biochemical pathways while also reporting on environmental effects [171]. Multiple studies have characterized the metabolomic profile of tissue, blood and urine samples to identify the distinct metabolic signature between human MIUC patients and healthy controls (summarized here) [172]. These methods could be potentially applied in real-time, during surgery or cystoscopy, to distinguish malignant from non-malignant surgical margins. Furthermore, the metabolomic signature of a tumor could potentially aid in tumor classification and “predict” whether a patient will respond to a specific treatment or is prone to developing metastases as disease progresses. For instance, the use of ultra-performance liquid chromatography-mass spectrometry (UPLC-MS) has shown that increased levels of phospholipids, ceramides and triglycerides in human UC cell lines was associated with cisplatin resistance [173]. Gas-liquid chromatography analysis in human MIUC identified an upregulation of oleic acid and stearic acid in human MIUC tissue versus normal tissue, while arachidonic acid and n-6 polyunsaturated fatty acids were more abundant in the adjacent normal bladder tissue [174].

Little work has been done in characterizing the metabolic signature of canine MIUC. However, in 2009, early studies used desorption electrospray ionization mass-spectrometry (DESI-MS) to identify different patterns in glycerophospholipid, sphingolipid and free fatty acids between malignant and adjacent normal canine urothelium of four dogs [175]. In 2012, Nuclear Magnetic Resonance (NMR) spectroscopy was used to compare the urinary metabolic profile of dogs with UC to those of healthy dogs. Choline, urea, methylguanidine, citrate, acetone and β-hydroxybutyrate were significantly upregulated between dogs with TCC and healthy dogs (*p* < 0.05) [176]. Furthermore, in 2018, invasive canine UC tumors were compared to normal canine bladder tissue without lower urinary tract disease using DESI-MS as well as the newer touch-spray mass-spectrometry (TS-MS) analyses to focus on fatty acid and phospholipid characterization. Results from both platforms identified oleic acid and the oleic acid dimer to be highly upregulated in invasive UC compared to normal bladder, similar to human studies mentioned above, whereas stearic acid showed the opposite pattern [177].

If dogs with MIUC present with non-specific clinical signs in the lower urinary tract, then a biomarker capable of distinguishing between malignant and non-malignant disease could lead to immediate treatment for patients with MIUC. Hence, metabolic analyses could provide great clinical benefit by identifying biomarkers capable of meeting these criteria. Our lab is currently focused on identifying urinary biomarkers by comparing samples from dogs with MIUC and other non-malignant diseases of the low urinary tract, such as urinary tract infection, stones, or proliferative urethritis. Finally, it should be highlighted that finding consensus between different metabolomic studies is challenging due to variation in sample sizes, clinical characteristics of the dog or human patients as well as advances in instrumentation over the past fifteen years.

## 8. Conclusions

Canine MIUC shares many similarities with human MIUC, including protein and gene homology, pathophysiological mechanisms of cancer initiation and progression, drug targets, drug resistance, and potential prognostic and diagnostic biomarkers. These parallels provide evidence for dogs being a naturally occurring and immunocompetent model for human MIUC in which experimental therapeutics could be tested. Thus, employing the principles of comparative oncology will enable the translation of molecular findings from dogs to humans and vice-versa.

## Figures and Tables

**Table 1:** Established and proposed therapeutic targets in canine MIUC.

Target	Drug
COX-1/ COX-2	Piroxicam, Meloxicam, Carprofen
COX-2	Firocoxib, Mavacoxib
COX- 5-LOX	Tepoxalin
DNA damage repair mechanisms	Cisplatin, Mitoxantrone, Doxorubicin
Microtubular proteins	Vinblastin
DNA synthesis	Gemcitabine
CCR4	Mogamulizumab
Survivin	EZN-3042
Pan- ErbB	Sapatinib
PDGFR, VEGFR, KIT, Flt3	SU11654
BRAF	Vemurafenib, Dabrafenib
Pan- RAF	LY3009120
MEK	Selumetinib, Trametinib
ERK	SCH772984
P-38	SB239063
JNK	SP600125
Nectin-4	rMV-SLAMblind

**Table 2:** Established and potential diagnostic and prognostic biomarkers for canine MIUC.

Biomarker	Method of Detection	Tissue/Biofluid	Function
p63	IHC	Tumor	Prognosis
Survivin (nuclear)	IHC	Tumor (↑) *	Diagnosis
Stratifin	IHC	Diagnosis	Diagnosis
uroplakin	IHC	Tumor (↓)	Diagnosis
	ELISA	Urine (↑)	
FGF	ELISA	Urine (↑)	Diagnosis
EGFR, HER-2	RT-qPCR	Tumor (↑)	Diagnosis
	IHC		
PDGFR-β, KIT	IHC	Tumor (↑)	Diagnosis
BRAF^V595E^	Droplet PCR	Urine (+) #	Diagnosis
	PCR	Plasma (+)	
Choline	NMR	Urine (↑)	Diagnosis
Urea	NMR	Urine (↑)	Diagnosis
Methylguanidine	NMR	Urine (↑)	Diagnosis
Citrate	NMR	Urine (↑)	Diagnosis
Acetone	NMR	Urine (↑)	Diagnosis
β-hydroxybutyrate	NMR	Urine (↑)	Diagnosis
Oleic acid	DESI-MS/ TS-MS	Tumor (↑)	Diagnosis
Stearic acid	DESI-MS/ TS-MS	Tumor (↓)	Diagnosis

* Increased levels might be diagnostic of MIUC, # Presence of the mutation might be diagnostic of MIUC. Abbreviations: FGF: fibroblast growth factor, EGFR: epidermal growth factor receptor, HER-2: human epidermal growth factor receptor 2, PDGFR: platelet-derived growth factor receptor, KIT: KIT- proto-oncogene receptor tyrosine kinase, ErbB: erythroblastic leukemia viral oncogene, Flt3: fms-like tyrosine kinase 3, BRAF: v-Raf murine sarcoma viral oncogene homolog B.
